# Serum and urine ^1^H NMR-based metabolomics in the diagnosis of selected thyroid diseases

**DOI:** 10.1038/s41598-017-09203-3

**Published:** 2017-08-22

**Authors:** Wojciech Wojtowicz, Adam Zabek, Stanislaw Deja, Tomasz Dawiskiba, Dorota Pawelka, Mateusz Glod, Waldemar Balcerzak, Piotr Mlynarz

**Affiliations:** 1 Bioorganic Chemistry Group, Department of Chemistry, Wroclaw University of Technology, Wroclaw, Poland; 2 First Department and Clinic of General, Gastroenterological and Endocrinological Surgery, Wroclaw, Poland; 3 Faculty of Chemistry, Opole University, Opole, Poland; 4 Department of Vascular, General and Transplantation Surgery, Wroclaw Medical University, Wroclaw, Poland

## Abstract

Early detection of nodular thyroid diseases including thyroid cancer is still primarily based on invasive procedures such as fine-needle aspiration biopsy. Therefore, there is a strong need for development of new diagnostic methods that could provide clinically useful information regarding thyroid nodular lesions in a non-invasive way. In this study we investigated ^1^H NMR based metabolic profiles of paired urine and blood serum samples, that were obtained from healthy individuals and patients with nodular thyroid diseases. Estimation of predictive potential of metabolites was evaluated using chemometric methods and revealed that both urine and serum carry information sufficient to distinguish between patients with nodular lesions and healthy individuals. Data fusion allowed to further improve prediction quality of the models. However, stratification of tumor types and their differentiation in relation to each other was not possible.

## Introduction

Despite the fact that algorithm for managing nodular thyroid diseases is well established the diagnosis remain challenging and as a result only 10–40% of surgically resected thyroid nodules are malignant^1^.

Thyroid lesions occur frequently in the adult population, with up to 7% having palpable nodules and 50–67% having nodules that are detected in ultrasound examination or autopsy^2–5^. Although less than 10% of thyroid lesions are cancerous, the prevalence of individual nodules or multinodular goiters makes thyroid cancer the most common endocrine malignancy^6–9^. American Cancer Society estimated the total number of new thyroid cancer cases in the United States will exceed 64,000 in 2016^10^, and an increasing number of new cancers detected each year is observed worldwide^11^.

The method of choice for the diagnosis of thyroid nodules is fine-needle aspiration (FNA) biopsy under ultrasound control followed by cytological evaluation^12–14^. Based on the recent Bethesda System for Reporting Thyroid Cytopathology, the FNA results are divided into 6 categories: nondiagnostic or unsatisfactory (I), benign (II), atypia of undetermined significance (AUS) or follicular lesion of undetermined significance (FLUS) (III), follicular neoplasm (FN) or suspicious for FN (IV), suspicious for malignancy (V), and malignant (VI)^15–17^. Nondiagnostic or unsatisfactory results of thyroid biopsy (Bethesda system category I) should ideally be limited to no more than 10% of the cases; however, according to some statistical studies, it occurs in up to 20% of specimens^6–21^. There is a need for repeated FNA in such situations^6, 15, 21^. Such a procedure is also recommended in patients with the FNA finding of AUS/FLUS (Bethesda system category III); however, surgery is also a management option here^6, 15, 21–23^. The treatment of choice in situations of Bethesda categories IV - VI is operation, although the risk of malignancy is above 97% only in category VI, while it is 60–75% in category V and only 15–30% in category IV^6, 15, 21^. Considering the frequency of nondiagnostic or unsatisfactory results of a thyroid biopsy but also the fact that AUS/FLUS diagnosis represents approximately 10% of FNA cases, 10% of FN or suspicious for FN cases, and almost 3% of suspicious for malignancy category^21^, it must be clearly stated that many invasive procedures in nodular thyroid disease are performed because of the lack of other diagnostic methods that could exclude malignancy. Therefore, there is a strong need for development of new diagnostic methods that could provide clinically useful information regarding thyroid nodular lesions in a non-invasive way. Discovery of such a method could prevent unnecessary repeated biopsies and surgical procedures like thyroid lobectomy or thyroidectomy.

Years of experience have shown the usefulness and the limitations of immunocytochemical analyses and genetic tests of material obtained by FNA as a supplement to cytology analysis. The evaluated markers have included galectin-3, E-cadherin, fibronectin, CD44v6, thyroid transcription factor 1, Cbp/p300-interacting transactivator 1, thyroglobulin, calcitonin, CEA, p27, cyclin D1, cytokeratin 19, thyroid peroxidase, HBME-1, beta-catenin, and p53 as well as searching for BRAF or RAS mutations and RET/PTC or PAX8/PPARɤ translocations^24–28^. The last four appear to have the greatest potential in differential diagnosis^29–32^. At the same time, no breakthrough in the process of thyroid malignancy diagnostics was achieved in the blood analysis, when using some of the aforementioned markers. The evaluated markers were, for example, galectin-3 and cytokeratin 19, as well as others such as metalloproteinase-1, chitinase 3-like 1 or angiopoietin-1^33^. Currently, the expectations are especially high with regard to the evaluation of microRNA (miRNA) serum profiles, although further studies are necessary^34–36^. It should be noted that to date, none of the described methods revolutionized the methodology for nodular thyroid disease diagnostics.

Metabolomics analysis of tissue samples demonstrated a potential for discrimination between nodular lesions and healthy thyroid as well as in grading malignant and benign lesions^37, 38^. Unfortunately, an invasive procedure is necessary to obtain the material for analysis, and thus other biomarkers need to be discovered. In this study we describe ^1^H NMR spectroscopy based metabolomic analysis of biofluids, which could improve the diagnostics of nodular thyroid disease in a non-invasive manner. The data was obtained by measurement of paired serum and urine samples from patients classified to the following four groups: HC – healthy control, NN – non-neoplastic nodules, AF – follicular adenoma and TC – papillary thyroid cancer.

## Methods

### Sample collection

Serum and urine samples were collected from patients operated on at the First Department and Clinic of General, Gastroenterological and Endocrinological Surgery of Wroclaw Medical University. The protocol for this study was approved by the Commission of Bioethics at Wroclaw Medical University (Approval no. KB-248/2010), and written informed consent was obtained from all of the patients before enrollment in the study. All of the patients were euthyroid and had a normal level of thyroid stimulating hormone (TSH). They were not treated with any drugs before the surgery. Serum and urine were sampled from the participating 67 subjects (Table 1).

**Table 1:** Patients demographic data. N – number of patients, f – female, m – male, age including standard deviation.

Type	N (f/m)	Age (±SD)
Non-neoplastic nodules (NN)	20(17/3)	56.1 ± 13.7
Follicular adenoma (FA)	13(12/1)	54.8 ± 21.1
Papillary thyroid cancer (TC)	17(17/0)	46.9 ± 15.5
Healthy Control (HC)	17(9/8)	39.2 ± 14.5

Serum was sampled from the peripheral vein from all of the participants after overnight fasting, and it was collected using serum tubes that were then centrifuged at 1000 x rpm for 15 minutes at 4 °C. The samples were stored in Eppendorf type tubes and kept at −80 °C until the analysis.

Urine samples (morning, first pass) were collected in urine test cups. They were centrifuged at 4000 rpm for 10 min, and then, the urine samples were stored in 2-mL aliquots in falcon tubes at −80 °C until further use.

### Sample preparation for NMR spectroscopy

Prior to the metabolomic experiment, the serum samples were thawed at room temperature,vortexed and 200 μL of serum was mixed with 400 μL of saline solution (0.9% NaCl, 15% D_2_O, containing 3 mM TSP). After centrifugation (12 000 x rpm, 10 min), 550 μL of supernatant was transferred to a 5-mm NMR tube. The samples were kept at 4 °C until measurement.

All of the urine samples were thawed at room temperature and mixed using a vortex mixer. The samples were centrifuged (12 000 x rpm, 10 min) and 400 μL of supernatant was mixed with 200 μL of PBS (0.5 M, pH = 7.00, 33% D_2_O, containing 3 mM NaN_3_ and 3 mM TSP). The samples were mixed again, and finally, an aliquot of 550 μL was transferred into a 5-mm NMR tube.

### ^1^H NMR measurements

The NMR spectra of the serum and urine samples were recorded at 300 K using an Avance II spectrometer (Bruker, GmBH, Germany) that was operating at a proton frequency of 600.58 MHz. The NMR spectra of the serum samples were recorded by using a CPMG pulse sequence with water presaturation (*cpmgpr1d* in Bruker notation). For each sample, 128 subsequent scans were collected with a spin-echo delay of 400 μs; there were 80 loops, a relaxation delay of 3.5 s, an acquisition time of 2.73 s, a time domain of 64 k, and a spectral width of 20.01 ppm.

The NMR spectra of the urine were recorded using NOESY pulse sequence with a water presaturation (*noesy1dpr* in Bruker notation) relaxation delay of 3.5 s, an acquisition time of 1.36 s, 128 transients, a time domain of 64 k, and a spectral width of 20.01 ppm.

The spectra were processed with a line broadening of 0.3 Hz and were manually phased and baseline corrected using Topspin 1.3 software (Bruker, GmBH, Germany). Serum spectra were referenced to an α-glucose signal (δ = 5.225 ppm), while urine spectra to the TSP resonance (δ = 0.000 ppm). Signal alignment was carried out using the correlation optimized warping algorithm (COW)^39^ and the *icoshift* algorithm implemented in Matlab (v 8.3, Mathworks Inc.)^40^. The water spectrum region was removed from the calculations. All of the spectra were normalized using the Probabilistic Quotient Normalization (PQN) method^41, 42^.

### Preprocessing of variables prior to analysis

The metabolite resonances were identified according to the assignments published in the literature and on-line databases (Biological Magnetic Resonance Data Bank and Human Metabolome Data Base). For quantification purposes, integrals of the non-overlapping signal fragments were used. All of the variables (originating from different fluids) were scaled by unit variance.

### Data fusion

The relative integrals of the resonances signals obtained from paired serum and urine samples data matrices were combined into one fusion data matrix. For the purpose of the model calculations, the names of the metabolites identified in the urine were replaced with structure - metabolite_[s] for serum and metabolite_[u] for urine to overcome overlaying metabolites. All repetitive metabolites from serum and urine were treated as separate variables.

### Multivariate data analysis

Multivariate data analysis was performed using SIMCA software (v 14.0, Umetrics). The order of the samples in the dataset was randomized. The discriminant version of the Partial Least Squares regression (PLS-DA) with a default k-fold cross validation procedure was used to determine the differences between the groups.

Samples were split into two datasets (model and test) based on the Kennard and Stone algorithm and randomized.

To improve the obtained models, variable selection using the VIP-plots with a jack-knifed confidence interval and confidence level of 0.95 was conducted. The variables that had a value VIP of below 0.8 were removed from the subsequent analysis until they had a negative influence on the R^2^ and Q^2^ parameters of the model. The new models were re-built on the basis of the selected variables, and then, the models’ reliabilities were tested with CV-ANOVA at the level of significance of *p* < 0.05.

The prediction performance of the VIP-PLS-DA models was estimated based on receiver operating characteristic (ROC) curves and area under curve (AUC) values. For this purpose, a *perfcurve* function from the Matlab statistical tool-box (Matlab v. 8.3, Mathworks, Inc.) was adopted. The specificity and sensitivity were determined according to the sample class prediction using the 7-fold cross-validated predicted values of the fitted *Y*-predcv (implemented in SIMCA-14 software) for observations in the model.

### Statistical data analysis

For each metabolite of the serum and urine samples, the percentage difference (PD) and relative standard deviation (RSD) were calculated using STATISTICA 12. The percentage difference was calculated based on the mean values of relative signal integrals in each group. The calculations were performed from left to right. For the chosen metabolites, the statistical significance based on the Mann–Whitney–Wilcoxon (p < 0.05) or Student t test (p < 0.05) was calculated.

## Results and Discussion

### Multivariate analysis in the diagnosis of thyroid lesions

Each pathological state, even at the cellular level, should be reflected in the body fluids, at least in the most abundant urine and blood, which can be less-invasively collected^43^. The changes that occur on a molecular basis in the thyroid tissue at the genomic and proteomic levels ought to be reflected in a variation in the metabolome profile of the biofluids. There, a specific variation in the homeostatic concentration of low-molecular compounds is expected to occur as a characteristic of the existing pathological conditions of thyroid gland^43^. In this work, for the first time, we have investigated paired urine and blood serum samples by the use of NMR methodology for healthy controls (HC) and patients who suffer from benign changes as well as those with advanced carcinogenesis.

**Figure 1:**
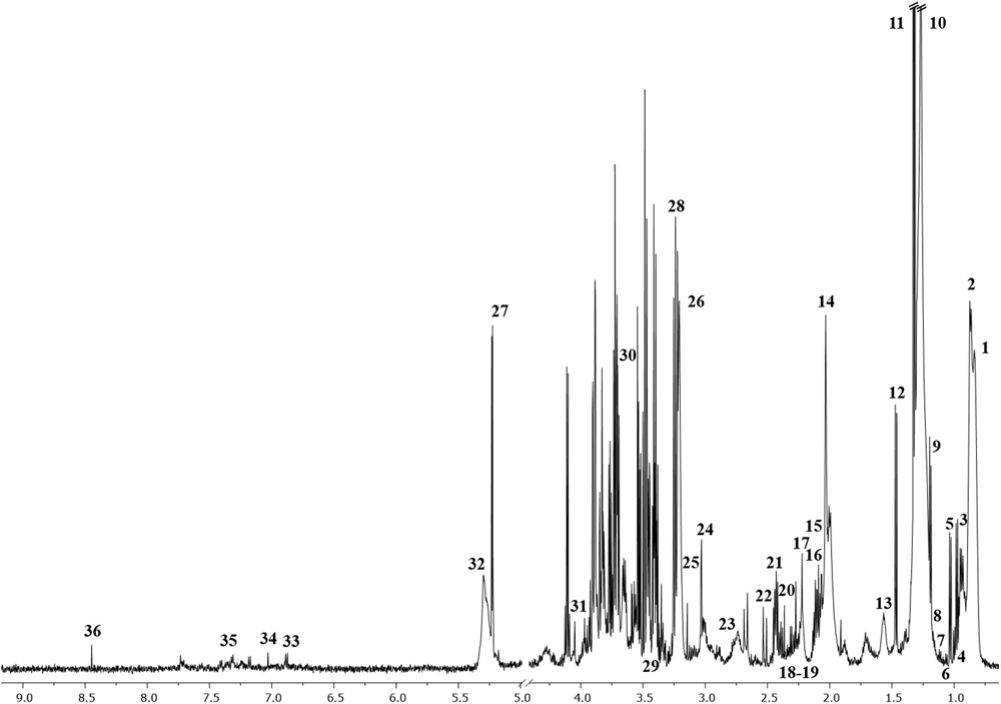
The representative spectrum ^1^H NMR obtained from serum samples of HC subjects. The following metabolites are identified: 1, L_1; 2, L_2; 3, Isoleucine; 4, Leucine; 5, Valine; 6, Unk_1; 7, 3-Hydroxybutyrate; 8, L_3; 9, L_4; 10, L_5; 11, Lactate; 12, Alanine; 13, L_6; 14, Acetate; 15, L_7; 16, L_8; 17, NAC; 18, Acetone; 19, Acetoacetate; 20, Pyruvate; 21, Glutamine; 22, Citrate; 23, Unk_2; 24, Creatine; 25, Dimethyl sulfone; 26, Chol+GPC+APC; 27, Glucose; 28, Betaine; 29, Methanol; 30, Glycerol; 31, Creatinine; 32, L_9; 33, Tyrosine; 34, π-Methylhistidine; 35, Phenylalanine; 36, Formate.

**Figure 2:**
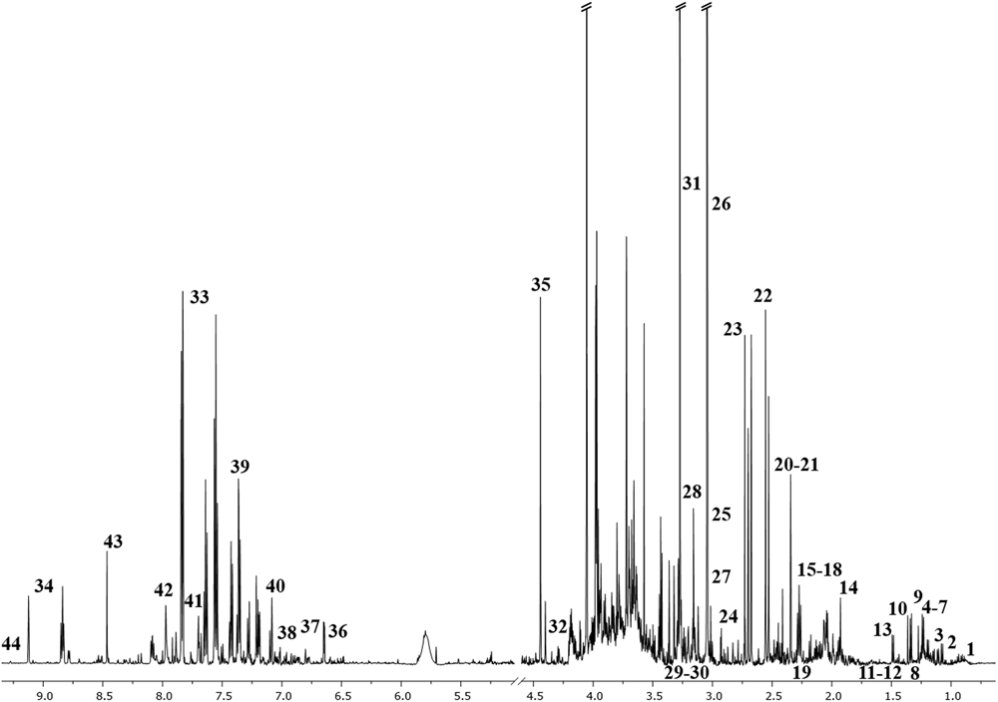
The representative spectrum ^1^H NMR obtained from the urine samples of HC subjects. The following metabolites are identified: 1, Unk_1; 2, 3-Hydroxyisobutyrate; 3, 3-Methyl-2-oxovalerate; 4, Isopropanol; 5, 3-Hydroxybutyrate; 6, Methylmalonate; 7, Fucose; 8, 3-Hydroxyisovalerate; 9, Lactate; 10, 2-Hydroxyisobutyrate; 11, 2-Phenylpropionate; 12, Unk_2; 13, Alanine; 14, Acetate; 15, Unk_3; 16, Unk_4; 17, N-Isovaleroylglycine; 18, Acetone; 19, 2-Aminoadipate; 20, Unk_5; 21, Unk_6; 22, Citrate; 23, Dimethylamine; 24, Unk_7; 25, Creatine; 26, Creatinine; 27, Carnitine; 28, TMAO; 29, Unk_8; 30, Unk_9; 31, Glycine; 32, Glycylproline; 33, Hippurate; 34, Trigonelline; 35, Ascorbate; 36, 2-Furoylglycine; 37, 3-Hydroxyphenylacetate; 38, Tyrosine; 39, N-Phenylacetylglycine; 40, 3-Indoxylsulfate; 41, Unk_10; 42, Formate; 43, Unk_11; 44, 1-Methylnicotinamide.

The representative ^1^H NMR serum spectrum of 36 and urine spectrum of 44 assigned metabolites in HC subject are presented in the examples in Figs 1 and 2. The metabolite resonances were identified according to assignments published in the literature and on-line databases (http://hmdb.ca and http://www.bmrb.wisc.edu).

### Discrimination between controls and thyroid lesions

**Figure 3:**
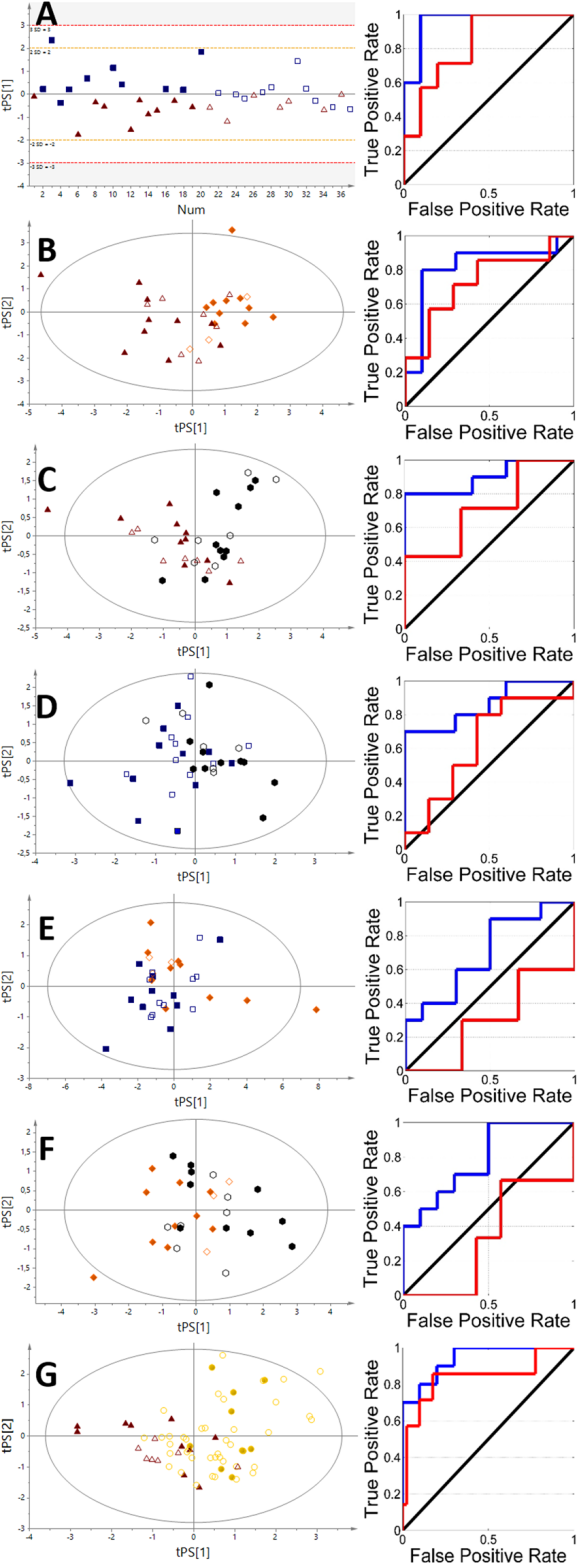
The VIP-PLS-DA models and ROC curves obtained from serum samples. (**A)** – NN vs HC; (**B)** – FA vs HC; (**C)** – TC vs HC; (**D)** – TC vs NN; (**E)** – FA vs NN; (**F)** – FA vs TC; (**G)** – P vs HC. Red triangles – healthy control; blue boxes – non-neoplastic nodules; yellow diamonds – follicular adenoma; black hexagons – papillary thyroid cancer; gold circles – patients. Solid symbols: training set; empty symbols: predicted test set.

**Figure 4:**
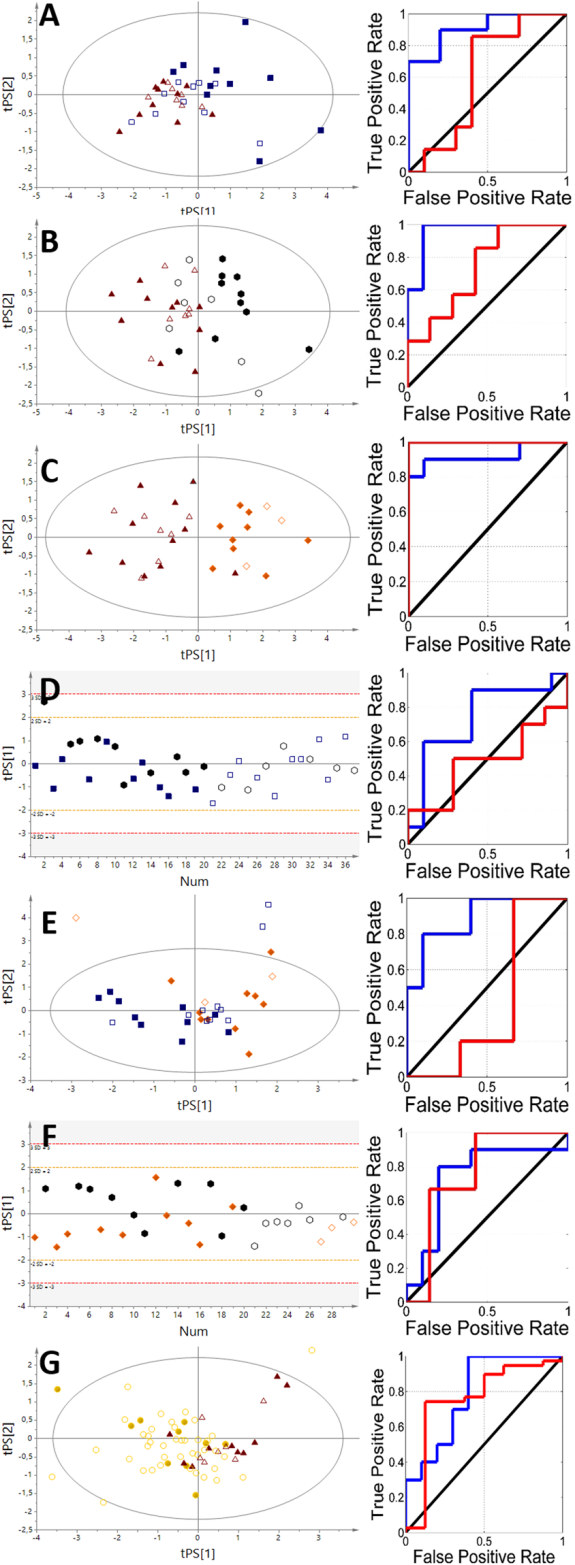
The VIP-PLS-DA models and ROC curves obtained from the urine samples. (**A)** – NN vs HC; (**B)** – FA vs HC; (**C)** – TC vs HC; (**D)** – TC vs NN; (**E)** – FA vs NN; (**F)** – FA vs TC; (**G)** – P vs HC. Red triangles – healthy control; blue boxes – non-neoplastic nodules; yellow diamonds – follicular adenoma; black hexagons – papillary thyroid cancer; gold circles – patients. Solid symbols: training set; empty symbols: predicted test set.

Initially, all of the obtained NMR data were subjected to calculations of seven discriminatory PLS models, for each type of thyroid lesion and each type of collected biological material. The selected metabolites were chosen based on the best parameters of separation between the groups by using the VIP scores, and they were used for further VIP-PLS-DA model calculations (Figs 3 and 4). The calculated parameters of the designed models as represented by ROC curves were compiled in Table 2.

**Table 2:** The parameters of PLS-DA models obtained from ^1^H NMR analysis of serum, urine samples and fusion data (*latent variables).

Comparison	Body fluid	AUC training set	AUC test set	R^2^X/R^2^Y	Q^2^(cum)	*p* value	Number of LV*
NN vs HC	SERUM	0.96	0.83	0.515/0.478	0.478	4.00E-03	1
FA vs HC		0.90	0.71	0.546/0.660	0.413	8.22E-02	2
TC vs HC		0.82	0.73	0.770/0.327	0.103	5.80E-01	2
TC vs NN		0.86	0.63	0.736/0.393	0.258	3.99E-01	2
FA vs NN		0.70	0.30	0.914/0.257	0.057	8.80E-01	2
FA vs TC		0.79	0.33	0.592/0.394	0.190	9.98E-01	2
P vs HC		0.94	0.84	0.618/0.575	0.415	8.00E-03	2
NN vs HC	URINE	0.91	0.61	0.534/0.574	0.312	3.96E-01	2
FA vs HC		0.92	1.00	0.445/0.629	0.442	9.64E-01	2
TC vs HC		0.96	0.73	0.608/0.622	0.459	1.28E-01	2
TC vs NN		0.74	0.49	1.00/0.244	0.123	3.26E-01	1
FA vs NN		0.89	0.40	0.565/0.508	0.343	1.47E-01	2
FA vs TC		0.74	0.76	1.00/0.257	0.134	2.94E-01	1
P vs HC		0.79	0.76	0.803/0.355	0.210	5.63E-01	2
NN vs HC	URINE SERUM FUSION	0.93	0.82	0.423/0.766	0.631	3.47E-04	2
FA vs HC		0.92	1.00	0.312/0.898	0.494	4.35E-02	2
TC vs HC		0.99	0.93	0.299/0.928	0.785	1.40E-06	2
TC vs NN		0.95	0.32	0.500/0.704	0.574	7.00E-04	2
FA vs NN		0.84	0.20	0.425/0.702	0.413	8.90E-02	2
FA vs TC		0.83	0.46	0.333/0.818	0.456	5.80E-02	2
P vs HC		0.97	0.95	0.280/0.857	0.500	3.00E-03	2

Each of analyzed biofluids exhibited different discrimination potential (Table 2). The best separation using serum between healthy subjects and patients was obtained for NN vs HC comparison (Q^2^ = 0.478, AUC test set = 0.83). The two other models, FA vs HC and TC vs HC, were on the same level of discrimination (AUC test set equal to 0.71 and 0.73) but did not pass the test of model significance. The urine models for these groups also showed a *p* value that was higher than 0.05, while the predictive potential between particular comparisons was in the following order: FA vs HC (AUC test set = 1) > TC vs HC (AUC test set = 0.73) > NN vs HC (AUC test set 0.61). Interestingly, in the comparison between healthy subjects and all of the collected thyroid lesions, All patients (P) vs HC were only slightly different between the biofluids, showing blood serum (AUC test set 0.84) to be more appropriate diagnostic material than urine (AUC test set 0.76). Conversely, the pairwise comparison between different thyroid lesions revealed that only one model (FA vs TC based on urine) provides satisfactory predictive power (AUC = 0.76).

**Figure 5:**
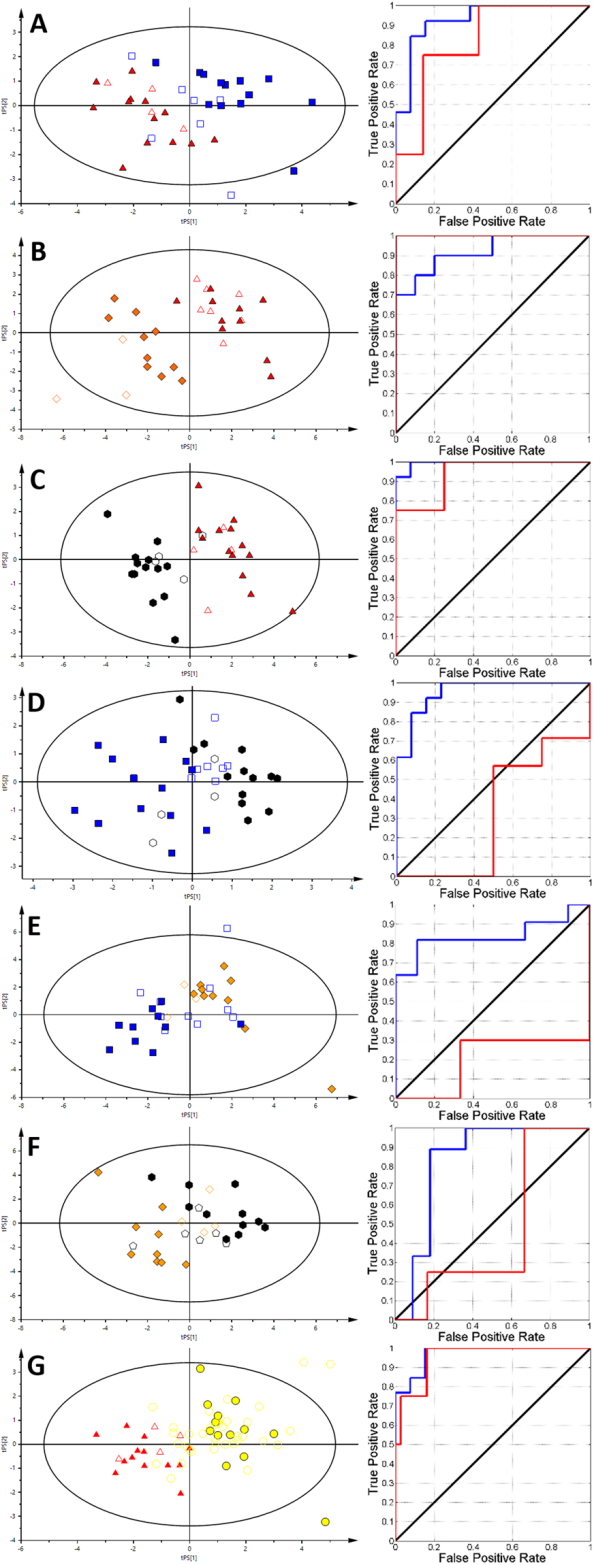
The VIP-PLS-DA models, ROC curves obtained from the fusion of data from urine and serum samples. (**A**) – NN vs HC; (**B)** – FA vs HC; (**C)** – TC vs HC; (**D)** – TC vs NN; (**E)** – FA vs NN; (**F)** – FA vs TC; (**G)** – P vs HC. Red triangles – healthy controls; blue boxes – non-neoplastic nodules; yellow diamonds – follicular adenoma; black hexagons – papillary thyroid cancer; gold circles – patients. Solid symbols: training set; empty symbols: predicted test set. Small violet circles – metabolites that influence group differentiations in PLS-DA models.

In the case of models obtained on the fusion data, the basic model parameters were significantly better (Table 2). In support of this finding, the results also showed differences in ROC curve (Fig. 5) and AUC training (Table 2) values for all of the comparisons, which indicates a better fit for the model that uses the selected samples in relation to the models, that were constructed separately on serum or urine. As in the case of the obtained models for each biofluid model based on data fusion, the best performance was observed in the case of the comparison of HC vs NN /FA/TC and the total number of patients (P) with pathological changes (Table 2). For all of the models, all of the values that were obtained were above 0.83 (FA vs TC), while the highest value was 0.99 for TC vs HC comparison. The possibility of formulating prediction models was also higher in terms of comparisons of HC to pathological changes of the thyroid (Table 2). The highest predictive value was characterized by FA vs HC, which reached the AUC test of 1.00, while the lowest value was obtained for NN vs HC with an AUC test of 0.82.

The data fusion from the serum and urine NMR measurements definitely strengthened of calculated models between the NN, FA, TC, P and the HC groups. That finding was due to the increased number of variables, which theoretically could add complementary information to the obtained models. The AUC values were between 0.82 and 1, which shows a high predictive potential based on combined information from both biofluids. Moreover, comparisons between different thyroid lesions were enhanced when data fusion was applied. Surprisingly, lesion development did not exhibit better predictive model abilities, as it has been previously found in the metabolomics investigation of thyroid tissues^37, 38, 44^. This fact might be explained by vascularization of the tumor tissue, where the size of the tumor could influence the type of vascularization^45^ and thus lead to providing less powerful information on the biochemical changes that are widely spread over the biological system.

### Metabolic differences in thyroid lesions

In our previous study, we showed that the differentiation of tumor type was possible by conducting aqueous tissue extracts^37^. Based on this findings we decided to investigate whether similar discrimination can be obtained using biofluids (individually or in combination), which unlike tissue biopsies, can be easily collected. Considering all low-molecular-weight compounds that were identified in NN vs HC, when comparing serum to tissue extract samples, only lactate and formate were statistically important in both studies and followed the same trend of increasing values of the relative integral in NN. However the tissue lactate was increasing systematically with NN > AF > TC, which was found to be reversal to blood serum level. In tissue, the lactate upregulation is associated with alanine and glucose increasing level, two main sources of it. While in blood serum these metabolites are only slightly changed, decreased alanine and increased glucose, but are not statistically significant. This might be evidence of its fast utilization from circulating blood as an answer for eg. energy demand. Similarly, formate strong upregulation was observed in blood serum and tissue however due to the high RSD it is hard at this stage to consider this molecule as a potential biomarker.

In urine and tissue extract only two metabolites were overlapping: 3-hydroxybutyrate (3-HB) and acetone, both statistically important and followed the same types of changes – decreasing in NN. Collectively in the serum and urine samples, 10 metabolites were statistically important in the NN vs HC comparison (Tables 3, 4).

**Table 3:** Significantly changed serum metabolites (^#^ - VIP plot selected metabolites; * - statistically significant metabolites).

Metabolites	Percentage difference							Relative Standard Deviation [%]				
	NN vs HC	FA vs HC	TC vs HC	FA vs NN	FA vs TC	TC vs NN	P vs HC	HC	NN	FA	TC	P
**Amino acids and derivatives**												
Isoleucine_[s]	0.7	−0.2	−1.3	−0.9	1.1	−2.1	0.2	9.2	7.4	8.7	6.2	9.2
Alanine_[s]	−2.8	−8.3	−11.9*^#^	−5.6	3.5^#^	−9.1	7.2	17.2	13.6	10.1	13	17.2
Betaine_[s]	−3.1	−4.2	−4.9	−1.2	0.7	−1.9	4	21.6	23.9	20.5	22.1	21.6
Creatine_[s]	8	8.4	16.7*	0.4	−8.3	8.7	−11.1*	23.2	12.9	14.8	13.2	23.2
Glutamine_[s]	−2.1	4.9	5.7	7.0*	−0.8	7.8*	−2.5	13.9	12.6	6.8	13.8	13.9
Leucine_[s]	−2.2	−6.1^#^	−7	−3.9	1	−4.9	4.8^#^	15.3	9.3	6.9	9.7	15.3
NAC_[s]	−8	7.9	−10.8	15.9^#^	18.6	−2.8	4.5	38.8	25.7	57.5	34.8	38.8
Tyrosine_[s]	−6.4	−18.1*^#^	−16.2*^#^	−11.7	−1.9	−9.9	12.6*^#^	23.9	20.6	19.5	15.7	23.9
Valine_[s]	−3.7	−13.6*^#^	−13.7*^#^	−9.9*^#^	0	−9.9*	9.6*^#^	19.6	11.8	8.4	14	19.6
π-Methylhistidine_[s]	−8.5	−10.1^#^	−5.6	−1.6	−4.5	2.9	7.9	16.1	13.2	11.8	14.7	16.1
**Carboxylic acids**												
Acetate_[s]	−2.9	−9.7	−16.8	−6.8	7.1	−13.9*^#^	9.2	40.3	17.8	25.5	18.7	40.3
3-Hydroxybutyrate_[s]	10.2	13	28.8	2.8	−15.9	18.7	−17.6	15.8	14.9	19	88.2	15.8
Acetoacetate_[s]	0.3	3.7	36.8	3.4	−33.2	36.4	−15.2	28	33.8	24.6	122.8	28
Citrate_[s]	14.1*^#^	13.2*^#^	8.7	−0.9^#^	4.4	−5.4	−12.1*	7.6	10.5	13	12.4	7.6
Formate_[s]	81.3*	85.5*	102.7*^#^	5.1	−22.1	27	−90.5*	103.8	85.4	94.6	101.2	103.8
Lactate_[s]	25.7*	17.5*^#^	−0.6	−8.2	18.2*^#^	−26.3*	−15.3	21.4	35.6	17.6	32.3	21.4
Phenylalanine_[s]	−1	−9.2^#^	−4.5	−8.2	−4.7	−3.5	4.3	14.7	12.2	14.8	17.5	14.7
Pyruvate_[s]	7.1	9.7^#^	−3	2.6	12.7^#^	−10.1	−4.5	20.5	28	32	25	20.5
**Cholines**												
Chol + GPC + APC_[s]	3	−1.1	5.2	−4.1	−6.2	2.1	−2.7	18.6	20.1	17.6	18.8	18.6
**Ketones**												
Acetone_[s]	−1.8	10.1	6.6	11.8	3.5	8.4	−4.3	44.3	24.4	47.6	69	44.3
**Sulfones**												
Dimethyl sulfone_[s]	0.4	−7.7^#^	−1.1	−8.1	−6.6	−1.5	2.2	28.5	29.2	36.1	22.9	28.5
**Monosaccharides**												
Glucose_[s]	7.9	7.4	10.8	−0.5	−3.5	2.9	−8.8*	17.1	14.9	10.7	17.1	17.1
**Alcohols**												
Methanol_[s]	−1.4	5.7	2.5	7.1	3.2	3.9	−1.8	22.6	18.5	17.3	19.1	22.6
Glycerol_[s]	9.0*	1.9	10.3*	−7.1*	−8.4^#^	1.2	−7.7*	13.4	8.4	9.7	14.6	13.4
**Imidazolinones**												
Creatinine_[s]	−12.1	−9.5	−18.6	2.6	9.1	−6.6	13.6*	15.3	31.7	20.5	13.7	15.3
**Unknown compounds**												
Unk_1_[s]	−33.5	−35.4	−12.5	−2	−23.1^#^	21.2*^#^	26.4	74.2	9.8	8.4	48.9	74.2
Unk_2_[s]	16.0*	7.4	3.1	−8.6	4.3	−12.9	−9.5	19.1	20.2	19.8	21.2	19.1
**Unknown lipids**												
L_1_[s]	3.7	0.2	3.8	−3.6	−3.6	0	−2.8	19.9	20	12.9	18.6	19.9
L_2_[s]	−10.7	6	−5.7	16.7^#^	11.8	4.9	4.4	37.4	18.4	37.4	29.1	37.4
L_3_[s]	3.3	0.5	0.1	−2.7	0.5	−3.2	−1.5	17	14.2	7.9	14.8	17
L_4_[s]	−5.6	1.5	−6.4	7.1^#^	7.9	−0.8	4	27.6	15.8	27.4	27.2	27.6
L_5_[s]	−20.6	−0.1	−14.9	20.5^#^	14.8	5.7	13	55.2	30.8	59.3	48.5	55.2
L_7_[s]	−5.5	0	−5.1	5.6^#^	5.1	0.5	3.9	20.1	10.8	21.7	18.8	20.1
L_8_[s]	0.5	2.8	3.7	2.3	−1	3.2	−2.2	11	11.6	15.3	11.4	11
L_9_[s]	−2.1	7.5	−3.3	9.6^#^	10.8	−1.2	−0.1	27.4	16.1	28	26.9	27.4

**Table 4:** Significantly changed urine metabolites (^#^ - VIP plot selected metabolites; * - statistically significant metabolites).

Metabolites	Percentage difference							Relative Standard Deviation [%]				
	NN vs HC	FA vs HC	TC vs HC	FA vs NN	FA vs TC	TC vs NN	P vs HC	HC	NN	FA	TC	P
**Amino acids and derivatives**												
2-Aminoadipate_[u]	−32.4	−25.3	−26.2	−7.2	−0.9	−6.3	−28.5	41.8	84.9	44.6	37.1	41.8
2-Furoylglycine_[u]	75.9^#^	93.8*^#^	116.4*^#^	−21.8	31.2	−52.1	93.3*	89	200	128	105	89
Alanine_[u]	2.5	1.6	−4.8	0.8	−6.5	7.3	−0.3	37.8	36	45	32.6	37.8
Creatine_[u]	5.9*	1	−6.3	4.9	−7.3	12.2	0.4	48.9	59.7	50.8	70.4	48.9
Glycine_[u]	−17.4	−7.2	10.1	−10.2	17.2	−27.4	−6.1	57.9	99.5	63.7	57.4	57.9
Glycylproline_[u]	10.8	7.8	8.7	3	0.9	2.1	9.3	33	24.2	33.1	20.1	33
*N*-Isovaleroylglycine_[u]	−19.2	43.9^#^	−25.9	−61.8	−67.9	6.8	−8.9	147	143	45	177	147
*N*-Phenylacetylglycine_[u]	3.6	−14.4	−5.7	18	8.8	9.3	−4.5	32.6	59.1	33.1	35	32.6
Tyrosine_[u]	19.7	30.5	−2.4	−10.9	32.8*^#^	22.2	14.2	56.1	47.1	46.4	35.1	56.1
**Carboxylic acids**												
3-Hydroxyisobutyrate_[u]	−20.4	−40.8	−56.4	20.8^#^	−16.5	37	−39.4^#^	36.5	63.1	142	111	36.5
2-Hydroxyisobutyrate_[u]	−33.8	−27.2*	−41.1*	−6.8	−14.3	7.5	−34.7*	27.5	59.2	32.8	46.3	27.5
2-Phenylpropionate_[u]	0.2	−143.5	−137.9*	143.7	11.2	138.0*	−117.2	50.9	173	189	162	50.9
3-Hydroxybutyrate_[u]	72.0*	−78.4*^#^	−54.2*	7.4	27.1	−19.7	−68.4*	86.3	81.1	90.2	75.4	86.3
3-Hydroxyisovalerate_[u]	5.8	29.2*^#^	−2.3	−23.5^#^	31.5*	8.2	8.4^#^	30.2	36.4	41.8	33.4	30.2
3-Hydroxyphenylacetate_[u]	49.5	−21.9	16.7	69.5*^#^	38.3	33.5	15.9	94.8	94.6	75.8	90.1	94.8
3-Methyl-2-oxovalerate_[u]	28.7	42.6*^#^	10.3	−14.3	−32.6	18.5	25.4	50.5	54.1	49	46.2	50.5
Acetate_[u]	−23.2^#^	−20.2	−25.5	−3	−5.4	2.3	−23.2	46.4	60.4	38.8	39.2	46.4
Citrate_[u]	−43.8*^#^	−27.3*^#^	−46.3*^#^	−17	−19.6	2.7	−40.7*	40.2	55.2	38.2	37.2	40.2
Formate_[u]	14.4	7.9	1.3	6.6	−6.5	13.1	8.1	69.1	72.2	56.3	53.2	69.1
Hippurate_[u]	67.2	2.7	29.7	64.8	27.1	39.4	34.7	134	46.1	131	140	134
Lactate_[u]	−18.7^#^	−12.3	−9.9	−6.5	2.4	−8.9	−14.1	37.5	51.3	43	33.8	37.5
Methylmalonate_[u]	37.8	24.1	0.9	14	−23.3	36.9	20.5	70.7	42.7	44.4	62.8	70.7
Trigonelline_[u]	47.7	23.1	41.3	25.3	18.6	6.7	38.7	79.4	88.8	121	72.9	79.4
**Amines**								0	0	0	0	0
Dimethylamine_[u]	12.5	15.1	17.7	−2.6	2.7	−5.3	14.9	58.6	38	23.1	28.2	58.6
TMAO_[u]	85.2	67.7	81.7	20.5	16.3	4.2	79.3	220	47.4	72.1	51.4	220
**Ketones**												
Acetone_[u]	−91.2*^#^	−67.7*^#^	−58.8*^#^	−27.8	9.9	−37.4	−75.8*	42.3	108	31.4	59.6	42.3
**Alcohols**												
Isopropanol_[u]	−74.4	−58	−68.8*	−18.4	−11.9	−6.5	−68.6	127	149	95.4	92.7	127
**Monosaccharides**												
Fucose_[u]	−3.9	−15.1	−15.1	11.2	0	11.2	−10.8^#^	43.3	44.1	33.9	52.7	43.3
**Imidazolinones**												
Creatinine_[u]	−7.3	−13.9	−2.9	6.6	11	−4.4	−7.6^#^	23.6	28.8	27.1	24.8	23.6
**Indoles**												
3-Indoxylsulfate_[u]	10.4	−24.4	−25.8^#^	34.6^#^	−1.4	36^#^	−12.5	59.9	69.3	41.8	54.8	59.9
**Pyridines and derivatives**												
1-Methylnicotinamide_[u]	20.1	19	7.6	1.1	−11.4	12.5	15.4	76.4	74.9	49.4	54.5	76.4
**Unknown compounds**												
Unk_1_[u]	−54.2	−16.2	16.8	−38.8	32.7	−69.4	−25.4	142	263	149	107	142
Unk_2_[u]	−50.5	−74.3*	−61.8	26.3^#^	14.1	12.3	−61.2	70.3	144	94.4	159	70.3
Unk_3_[u]	−14.5	−19.6*^#^	−21.8*	5.1	−2.3	7.4	−18.4*	21.9	29.8	16	23.3	21.9
Unk_4_[u]	−12.7*	−17.9*	−14.5*	5.2	3.4	1.8	−14.7*	14.8	19.1	14.4	18.3	14.8
Unk_5_[u]	−12.5	−54.8*	−10.3	43.1	45.1	−2.1	−25.1	46	83.4	63.1	81.7	46
Unk_6_[u]	−17.9	−10.1	−40.2	−7.9	−30.4	22.6	−24.4	67.5	114	65.9	68.2	67.5
Unk_7_[u]	−29.7	−17.4	−18.9	−12.5	−1.5	−11	−23	58.8	62.9	46.2	34.1	58.8
Unk_8_[u]	23.5	42.4	−1.3	−19.4	−43.6	24.8	18.6	80.9	105	134	73.5	80.9
Unk_9_[u]	−51.7^#^	−22	−36.5^#^	−30.6	−14.9	−15.9	−39.7	58.1	78.5	64.2	56	58.1
Unk_10_[u]	−19.2	−20.0*^#^	−15.4*	0.8	4.6	−3.8	−18.1*	19.9	26.5	12	18	19.9
Unk_11_[u]	80	79.8	−81.7*	0.2	2.3	−2.1	80.5*	91.5	188	188	169	91.5

In contrast, in the tissue study, 16 metabolites with significant changes were found^37^. In the assessment of changes in the metabolite statistical data of FA vs HC, for serum and urine samples, four metabolites and three metabolites, respectively, were matched to the tissue study results, namely, valine, citrate, lactate, and tyrosine for serum and citrate, acetone and 3-hydroxybutyrate for urine samples. In serum samples along the statistically important metabolites in both studies, valine and tyrosine percentage differences were decreased in FA, which was in contrast to the trend in the aqueous tissue extract study. However, the changes in tissue extracts of citrate (decreasing) and lactate (increasing) are of opposed direction, while in serum blood both metabolites are increased in comparison to HC group. This data can pronounce the different changes, which occurred at the local level (tissue) and whole metabolism as an answer for pathological state. Another example can be shifted balance of valine, where decreased level in blood serum is observed and increased in tissue extract. Additionally the decreased level of amino acids especially relative integral of serum tyrosine, could not only be related to protein biosynthesis but also for the synthesis catecholamines^46^.

In the assessment of changes in the urine samples, the decreased trend in citrate, acetone and 3-hydroxybutyrate level for the FA group were reported in both studies showed metabolism directed towards energy demand. In the tissue extract study, a total of 15 metabolites were statistically important, while overall for serum and urine, 17 compounds were found, where the majority belonged to the urine – 12 (Supplementary Tables S1 and S2).

The third comparison was the TC vs HC subjects. The differences between these most distant groups should have given the largest differences written in the molecular information. In the tissue extract study, 22 metabolites from 26 identified were statistically important, while surprisingly, in serum and urine collectively, only 17 were significantly changed. Only four of the identified metabolites from serum and two from urine matched the results from the previous study^37^. Valine, alanine, creatine and tyrosine in serum samples were decreasing in TC, whereas the aqueous tissue extract showed the opposite trend. Only the creatine, replenishing energy in ADP-ATP cycle metabolite, which increased level is observed in the TC group matched with the changes from the previous study. In urine, citrate and acetone were statistically important, and it followed the same decreasing trend as in the tissue extract. The distinguishing of HC among all of the other investigated thyroid lesions appears to be possible based on the serum and urine samples. However, the molecular composition outcome was not as good as from the aqueous tissue extract in the previous study. The consequence of having a lower quantity of statistically important metabolites obtained in this study was that discrimination of the thyroid nodules types was more difficult. Among all of the comparisons between each type of thyroid lesion, only two common metabolites were identified in serum, which matched with the results obtained from the aqueous tissue extracts. From the FA vs NN comparison – the valine relative integral was decreased for the FA group in both studies, in TC vs NN valine and lactate decreased in serum for the TC group, with the opposite trend in the tissue extract study. For the FA vs TC comparison – only serum lactate was decreased in the TC group, which is also in contradiction to the previous study^37^. An increased level of lactate in the processes of carcinogenesis is a common symptom and it was unexpected that the level is the highest in the NN group.

In conclusion no significant changes were observed in the total lipid profile. The acetone level identified in the urine samples followed exactly the same trend, which was recognized in the tissue extract samples study. Moreover, in most cases is positively correlated with its precursor 3-hydroxybutyrate (except tissue TC vs HC). This may indicate that the level of acetone in the urine samples may be prognostic factor for thyroid nodules. The increased level of lactate in blood and tissue with opposite direction of change in urine can be related to possible influence of hypoxia microenvironment^47^ occurring in tissue and its direct translation on blood serum^48^ (comparison NN, FA vs HC). The lack of lactate level changes in the comparison TC vs HC can be caused by strong local tissue changes and/or the limited flow between these two compartments.

Clearly, changes were much more pronounced in the tissue extracts then in serum or urine. This is however, to be expected as tissue collected *in situ* should reflect metabolic state of the lesion, than any of biofluids that reports whole organism metabolism.

All our findings appear to be rational combining three biological compartments, where the obtained data from the tissue extract samples were directly occurred in the place of major pathological disturbances. In the case of serum and urine samples, visible changes in the relative integral of the metabolites could be caused by the general response of the biological system for homeostasis disorder, which could be more subtle then the changes directly in the pathological tissue^37^.

## Conclusions

The models based on the fusion data have higher parameters and predictive potential compared with most of the models that were calculated separately for each body fluid. That finding indicates that combined datasets exhibit synergy that increases model stability and enhances diagnostic potential. However, it should also be noted that stratification of tumor types and their differentiation in relation to each other could not be obtained.

The VIP-PLS-DA method allows us to identify metabolites that are biomarker candidates and should be investigated in detailed in future.

Our study also allowed us to obtain a model with a 100% prediction for the FA vs HC comparison. The models that were calculated for comparisons between diseases units were of low quality, which could be connected to indications of similar changes in the distribution of metabolites in the organism. This similarity could negatively affect the creation of high-quality diagnostic models based on the proton NMR technique.

Despite the relatively good results in some comparisons, studies must be conducted on a larger cohort of patients in order confirm predictive potential of selected metabolites in diagnosing thyroid lesions.

## Declarations

### Ethics approval and consent to participate

The study was carried out in accordance with the Declaration of Helsinki. Serum and urine samples were collected from patients who were operated on at the First Department and Clinic of General, Gastroenterological and Endocrinological Surgery of Wroclaw Medical University. The protocol for this study was approved by the Commission of Bioethics at Wroclaw Medical University (Approval no. KB-248/2010).

### Consent for publication

All subjects read and signed a written informed consent prior the enrollment to the study.

### Availability of data and material

The datasets generated during and/or analyzed during the current study are available from the corresponding author upon request.

## Electronic supplementary material

Supplementary Information
