# Supplementary material for: Serum and urine 1H NMR-based metabolomics in the diagnosis of selected thyroid diseases
Source: Sci Rep. 2017 Aug 22;7:9108. doi: 10.1038/s41598-017-09203-3 (PMC5567318; doi:10.1038/s41598-017-09203-3)
Supplement: Supplementary file 1 — Supplementary Information [file 41598_2017_9203_MOESM1_ESM.doc]

**Serum and urine ^1^H NMR-based metabolomics in the diagnosis of selected thyroid diseases.**

**Authors:** Wojciech Wojtowicz^1^, Adam Zabek^1^, Stanislaw Deja^3^, Tomasz Dawiskiba^4^, Dorota Pawelka^2^, Mateusz Glod^2^, Waldemar Balcerzak^2^, Piotr Mlynarz*^1^

^1^ Bioorganic Chemistry Group, Department of Chemistry, Wroclaw University of Technology, Wroclaw, Poland

^2^ First Department and Clinic of General, Gastroenterological and Endocrinological Surgery, Wroclaw, Poland

^3^ Faculty of Chemistry, Opole University, Opole, Poland

^4^ Department of Vascular, General and Transplantation Surgery, Wroclaw Medical University, Poland

**Table S1** Significantly changed metabolites in both studies (HC vs thyroids changes). (* statistically significant metabolites, **Results from Deja S. et al. Follicular Adenomas Exhibit a Unique Metabolic Profile. 1H NMR Studies of Thyroid Lesions, 2013)

| Metabolite | Percentage difference | | | | | | | | |
| --- | --- | --- | --- | --- | --- | --- | --- | --- | --- |
|  | Tissue** | | | Serum | | | Urine | | |
|  | NN vs H | FA vs HC | TC vs H | NN vs H | FA vs HC | TC vs H | NN vs H | FA vs HC | TC vs H |
| Isoleucine | 4.3 | 76.7* | 73.8* | 0.7 | -0.2 | -1.3 | - | - | - |
| 2-Hydroxyisobutyrate | - | - | - | - | - | - | -33.8 | -27.2* | -41.1* |
| 3-Hydroxybutyrate | -20.4* | -32.2* | 5.9 | 10.2 | 13 | 28.8 | 72.0* | -78.4* | -54.2* |
| Acetate | 23.0a | 16.1 | 27.9* | -2.9 | -9.7 | -16.8 | -23.2 | -20.2 | -25.5 |
| Acetone | -30.2* | -36.5* | -39.2* | -1.8 | 10.1 | 6.6 | -91.2* | -67.7* | -58.8* |
| Alanine | 19.1* | 81.7* | 126.6* | -2.8 | -8.3 | -11.9* | 2.5 | 1.6 | -4.8 |
| Choline | 63.4* | 13.8 | 72.2* | - | - | - | - | - | - |
| Citrate | -24.8 | -66.5* | -85.4* | 14.1* | 13.2* | 8.7 | -43.8* | -27.3* | -46.3* |
| Creatine | 2.4 | 24.8 | 39.2* | 8 | 8.4 | 16.7* | 5.9* | 1 | -6.3 |
| Formate | 48.9* | -1.3 | 21.9 | 81.3* | 85.5* | 102.7* | 14.4 | 7.9 | 1.3 |
| Glutamate | 53.4* | 57.6* | 103.9* | - | - | - | - | - | - |
| Glycerol | - | - | - | 9.0* | 1.9 | 10.3* | - | - | - |
| Glycine | 33.4* | 60.9* | 81.2* | - | - | - | -17.4 | -7.2 | 10.1 |
| Histidine | 34.7* | 11.4 | 81.4* | - | - | - | - | - | - |
| Hypoxanthine | 41.1* | 47.5* | 49.4* | - | - | - | - | - | - |
| Lactate | 71.9* | 100.2* | 187.8* | 25.7* | 17.5* | -0.6 | -18.7 | -12.3 | -9.9 |
| Methionine | 32.4* | 37.6* | 82.5* | - | - | - | - | - | - |
| Myo-inositol | 10.7 | -10.8 | -49.1* | - | - | - | - | - | - |
| NAC | -7.7b | -38.6* | -34.3* | -8 | 7.9 | -10.8 | - | - | - |
| PC | 35.0* | 25.8 | 30.4 | - | - | - | - | - | - |
| Phenylalanine | 24.8* | 63.6* | 99.1* | -1 | -9.2 | -4.5 | - | - | - |
| Scyllo-inositol | 48.1 | -3.3 | -63.7* | - | - | - | - | - | - |
| Succinate | 39.7* | 71.5 | 63.0* | - | - | - | - | - | - |
| Taurine | 14.4b | 24.6 | 44.7* | - | - | - | - | - | - |
| Tyrosine | 31.5* | 75.7* | 87.8* | -6.4 | -18.1* | -16.2* | 19.7 | 30.5 | -2.4 |
| Uracil | 35.8 | 58.2 | 72.3* | - | - | - | - | - | - |
| Valine | -1.9 | 83.8* | 104.8* | -3.7 | -13.6* | -13.7* | - | - | - |
| β-glucose | 76.0* | 162.0* | 48 | 7.9 | 7.4 | 10.8 | - | - | - |

**Table S2**. Significantly changed metabolites in both studies (thyroids changes vs thyroids changes). (* statistically significant metabolites, **Results from Deja S. et al. Follicular Adenomas Exhibit a Unique Metabolic Profile. 1H NMR Studies of Thyroid Lesions, 2013)

| Metabolite | Percentage difference | | | | | | | | |
| --- | --- | --- | --- | --- | --- | --- | --- | --- | --- |
|  | Tissue | | | Serum | | | Urine | | |
|  | FA vs. NN | FA vs TC | TC vs NN | FA vs. NN | FA vs TC | TC vs NN | FA vs. NN | FA vs TC | TC vs NN |
| Alanine | 52.6* | -24.7 | 90.3* | -5.6 | 3.5 | -9.1 | 0.8 | -6.5 | 7.3 |
| Choline | -30.4 | -51 | 5.4 | - | - | - | - | - | - |
| Citrate | -55.4* | 56.5* | -80.6* | -0.9 | 4.4 | -5.4 | -17 | -19.6 | 2.7 |
| Creatine | 21.9 | -11.6 | 36.0* | - | - | - | 4.9 | -7.3 | 12.2 |
| Glutamine | - | - | - | 7.0* | -0.8 | 7.8* | - | - | - |
| Glycine | 20.6 | -12.6 | 35.9* | - | - | - | -10.2 | 17.2 | -27.4 |
| GPC | 16.9 | 25.8* | -13.3 | - | - | - | - | - | - |
| Histidine | -17.3 | -62.8* | 34.6* | - | - | - | - | - | - |
| Isoleucine | 69.4* | 1.7 | 66.6* | -0.9 | 1.1 | -2.1 | - | - | - |
| Lactate | 16.5 | -43.7* | 67.4* | -8.2 | 18.2* | -26.3* | -6.5 | 2.4 | -8.9 |
| Methionine | 3.9 | -32.6* | 37.8* | - | - | - | - | - | - |
| Myo-inositol | -19.4 | 42.9* | -54.0* | - | - | - | - | - | - |
| NAC | -33.5* | -7 | -28.8* | 15.9 | 18.6 | -2.8 | - | - | - |
| Phenylalanine | 31.1 | -21.7 | 59.5* | -8.2 | -4.7 | -3.5 | - | - | - |
| Scyllo-inositol | -34.7 | 62.4* | -75.5* | - | - | - | - | - | - |
| Taurine | 9 | -16.1 | 26.5* | - | - | - | - | - | - |
| Tyrosine | 33.6 | -6.9 | 42.8* | -11.7 | -1.9 | -9.9 | -10.9 | 32.8* | 22.2 |
| Valine | 87.4* | -11.4 | 108.8* | -9.9* | 0 | -9.9* | - | - | - |
